# Cost-effectiveness of abobotulinumtoxinA plus best supportive care compared with best supportive care alone for early treatment of adult lower limb spasticity following an acute event

**DOI:** 10.1371/journal.pone.0296340

**Published:** 2024-02-01

**Authors:** Peter Moore, Natalya Danchenko, Diana Weidlich, Alejandra Rodarte Tijerina

**Affiliations:** 1 Market Access Australia and New Zealand, Ipsen, Melbourne, VIC, Australia; 2 Global Market Access and HEOR, Ipsen, Boulogne-Billancourt, France; 3 Evidence, Value, and Access Consulting, Clarivate, Munich, Germany; 4 Evidence, Value, and Access Consulting, Clarivate, London, United Kingdom; The University of Sydney, AUSTRALIA

## Abstract

**Objectives:**

Spasticity is an incurable chronic condition, and patients with spasticity frequently experience symptoms such as muscle stiffness, restricted mobility, fatigue, spasms, and pain. The study objective was to assess the cost-effectiveness of abobotulinumtoxinA plus best supportive care compared with best supportive care alone for the early treatment of adult lower limb spasticity following an acute event (e.g. stroke or traumatic brain injury), from an Australian payer perspective.

**Methods:**

Using clinical data from published pivotal trials, an economic model based on a Markov model was developed to capture changes in treatment costs, healthcare resource use costs, functional outcomes, and health-related quality of life over a lifetime horizon. Scenario analyses and a probabilistic sensitivity analysis were conducted to explore the uncertainty in the model parameters and assumptions used in the base case.

**Results:**

AbobotulinumtoxinA plus best supportive care was cost-effective versus best supportive care, yielding an incremental cost-effectiveness ratio of $35,721 per quality-adjusted life year gained. Sensitivity analyses confirm the robustness of the base case, with most results remaining below the commonly acceptable cost-effectiveness willingness-to-pay threshold of $75,000 per quality-adjusted life year for cost-effectiveness in Australia. Inputs and assumptions that produced the top four highest incremental cost-effectiveness ratios include the application of different health resource utilisation source, short time horizon, unweighted regression analyses to determine regression probabilities, and no stopping rule. AbobotulinumtoxinA plus best supportive care has a 74% probability of being cost-effective compared with best supportive care alone at the willingness to pay threshold.

**Conclusion:**

AbobotulinumtoxinA plus best supportive care treatment is cost-effective in Australia for the management of adult lower limb spasticity in patients treated within 2 years of an acute event.

## Introduction

Spasticity is an incurable chronic condition that causes deformity and pain, and usually occurs from an acute event, such as a brain injury (due to stroke, trauma, hypoxia, injection or surgery) or spinal cord injury (SCI), but may also result from neurodegenerative aetiologies such as multiple sclerosis or cerebral palsy [1, 2]. A breakdown of motor responses to sensory input as a result of damage to the brain or spinal cord leads to hyper-excitability of the segmental central nervous system [3]. Patients with spasticity frequently experience symptoms such as muscle stiffness, restricted mobility, fatigue, spasms, and pain [4–7].

Adult lower limb spasticity (ALLS) presents in the hip, knee, ankle, or foot [8]. Abnormal limb posturing that develops in patients with lower limb spasticity includes equinovarus foot, flexed knee, adducted thighs, and flexed hip, and all of these deformities may impede activities of daily living such as bed positioning, sitting balance, chair-level activities, transfers, and standing up [9].

The incidence and prevalence of ALLS varies by geographical region. However, incidence values as reported by condition in literature includes: an annual incidence of 30–485, 100–235, and 0.2–8 per 100,000 for stroke, traumatic brain injury (TBI), and SCI, respectively [8]. Furthermore, prevalence values for stroke and SCI are reported as 40–600, and 22–90 per 100,000, respectively [8]. The prevalence of severe focal spasticity following an acute event is estimated at approximately 270 and 106 per 100,000 relating to stroke and TBI, respectively. Additionally, approximately 33% of patients with stroke and 75% of patients with TBI will develop spasticity that requires treatment [10].

Published guidelines focus on the promotion of management of spasticity due to neurological illness or injury [10]. The primary aim for the treatment of ALLS following an acute event is to maintain length and allow normal positioning of the limbs to prevent secondary soft tissue shortening and increase movement. Physical therapy forms the basis of the treatment recommended for spasticity [10]. Also recommended in the guidance, is the administration of spasticity medication to complement physical therapy [10].

International and Australian guidelines agree with how the management of spasticity should be undertaken, and the clinical benefit of the spasticity medication, botulinum neurotoxin (BoNT-A), in treating patients with lower limb spasticity. The guidelines are consistent in their conclusions that BoNT-A improves muscle tone in patients with lower limb spasticity and is recommended as a treatment option for patients with ALLS [10–14]. Early intervention with BoNT-A leads to better clinical outcomes as it allows clinicians to take advantage of the plasticity of muscles before contracture of the muscles limits the functional improvements that may be achieved [15–18].

Currently in Australia, abobotulinumtoxinA (aboBoNT-A) (Dysport^®^) and onabotulinumtoxinA (onaBoNT-A) (Botox^®^) are medications approved for use in the treatment of ALLS, including the treatment of moderate to severe ALLS following an acute event such as a stroke, TBI, SCI, or cerebral hypoxia.

The efficacy of aboBoNT-A was evaluated in two pivotal randomised controlled Phase III trials (Study 140 [NCT01249404; a double-blind study] and 142 [NCT01251367; an open-label extension trial of study 140]) [19]. Both trials were conducted in ambulatory participants post stroke or TBI (18–80 years) with spastic hemiparesis causing gait dysfunction [19]. In the double-blind study, the primary objective was to demonstrate single aboBoNT-A injection (aboBoNT-A 1,000 U or 1,500 U) efficacy compared with placebo in the lower extremity, the primary population for efficacy analyses was all randomised participants who received one or more study medication injections (intent-to-treat [ITT] population), and the primary endpoint was change from baseline after 4 weeks in the gastrocnemius-soleus complex (GSC) muscle tone (Modified Ashworth Scale [MAS] knee extended) [19]. In total, 98.19% of the total population was composed of the ITT population [19]. Secondary endpoints included physician global assessment (PGA) score and change from baseline in 10-m comfortable barefoot walking speed without walking aids, and exploratory endpoints included the mean change from baseline in soleus muscle tone (MAS knee flexed), spasticity, range of active ankle dorsiflexion, and measured knee flexed and extended [19]. The primary and secondary objectives of the open-label study were to assess the long-term safety and long-term efficacy of aboBoNT-A injections, respectively, for ≤4 treatment cycles at ≥12 week intervals, over ≤18 months [19].

These trials demonstrated a significant reduction in muscle tone in the gastrocnemius and soleus muscles upon administration of aboBoNT-A versus placebo, and a long duration of action [19]. Mean (95% confidence interval) MAS GSC changes from baseline at week 4 were −0.5 (−0.7 to −0.4) (placebo, n = 128), −0.6 (−0.8 to −0.5) (aboBoNT-A 1,000 U, n = 125; p = 0.28 vs placebo), and −0.8 (−0.9 to −0.7) (aboBoNT-A 1,500 U, n = 128; p = 0.009 vs placebo) after a single treatment [19]. Upon combining data from all cycles, 15–32% of participants did not require reinjection at week 12 [19]. Early treatment initiation is the only treatment effect modifier included in the model.

AbobotulinumtoxinA is efficacious in the treatment of broader ALLS population, and early intervention can positively impact the lives of patients and reduce healthcare resource utilisation (due to the improvement in walking speed and the potential for less injections and associated healthcare visits, respectively). However, there is a paucity of information on the cost-effectiveness of this treatment as an early intervention in patients with ALLS. Therefore, the objective of this study was to assess the cost-effectiveness of aboBoNT-A plus best supportive care (BSC) compared with BSC alone for the treatment of ALLS following an acute event occurred in less than 2 years before treatment initiation, from the perspective of the Australian healthcare system.

## Methods

### Model overview

A cost-utility analysis was conducted to assess the cost-effectiveness of aboBoNT-A plus BSC compared with BSC alone for the early treatment of ALLS following an acute event (stroke or TBI) in Australia. Here, BSC (physical/occupational therapy alone/oral therapy) is commonly applied alone as standard of care for these patients and therefore it was selected as a relevant comparator for this analysis. Comparison with the alternative treatment, onaBoNT-A, was not possible due to lack of head-to-head clinical trials comparing onaBoNT-A and aboBoNT-A in this indication due to studies seldom reporting walking speeds, and non-availability of reported outcomes in the onaBoNT-A trials that could be useful for an indirect comparison and an economic model.

**Fig 1:**
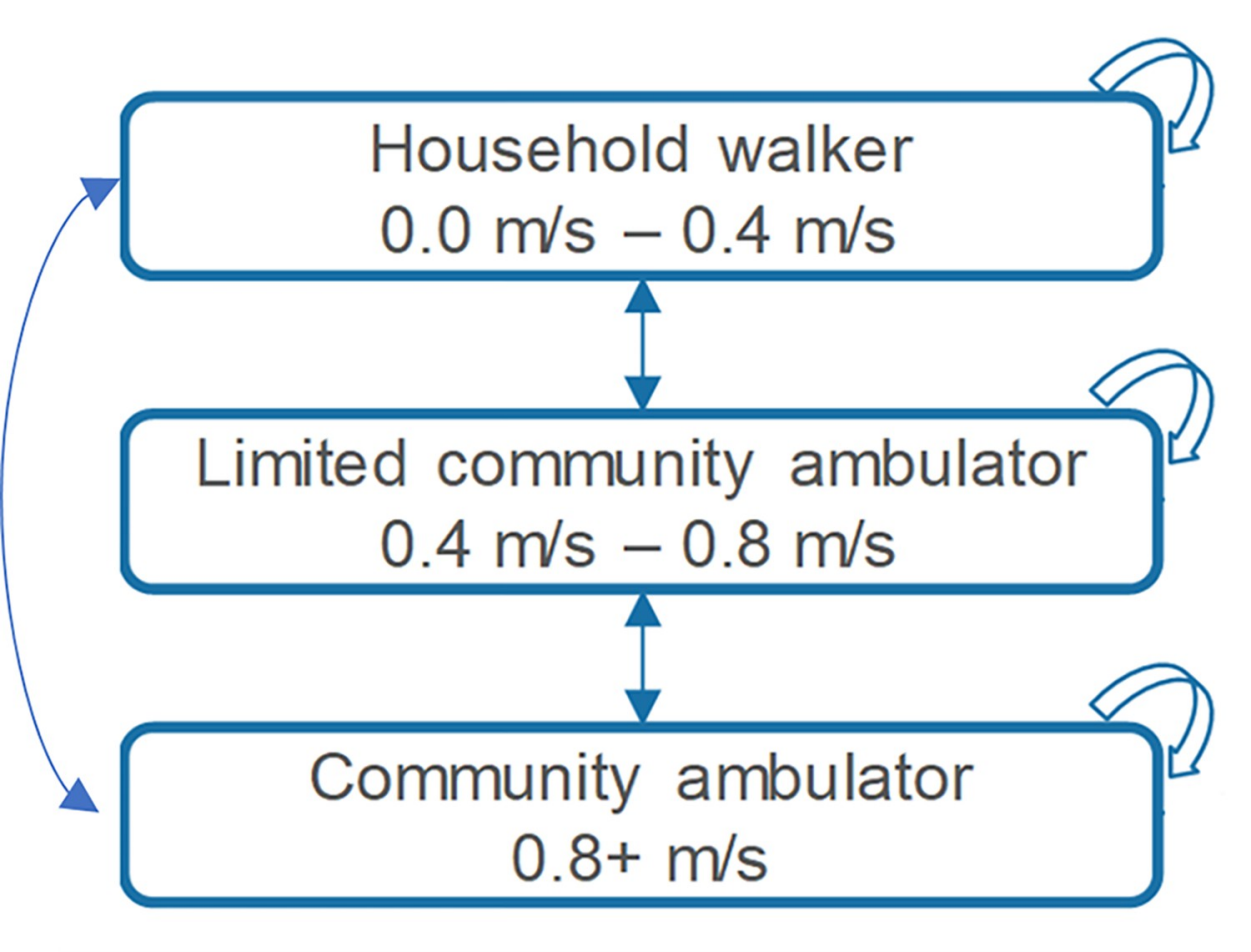
Schematic of the Markov model structure.

An economic model based on a Markov model was developed to capture changes in treatment costs, functional outcomes, HRU, and health-related quality of life (HRQoL) over time. A model diagram is presented in Fig 1.

Health states used in the model were defined based on walking speed classifications reported by Perry et al, 1995 [20]. Patients with spasticity frequently experience symptoms such as muscle stiffness and fatigue [4–7]; a reduction in walking speed can also result in stiffness and fatigue, and therefore can be used as a proxy measure to determine the health of the patient. Characterisation of the main health states experienced by patients was conducted using absolute walking speed levels; the ability of an individual to maintain functional walking performance can be predicted by their walking speed [20]. Absolute walking speed levels include community ambulator, limited community ambulator, and household walker.

The model considers a treatment discontinuation rate based on observations from the clinical trials which were linked to the occurrence of an adverse event causing the patient to stop their treatment. Additionally, reflecting Australian routine clinical practice, non-responders (defined as <0.13 m/s change in comfortable barefoot walking speed from baseline) after four 12-week treatment cycles were required to stop treatment with aboBoNT-A. Patients who discontinued treatment were assumed to lose treatment effect and were unable to return to treatment. Additionally, patients who discontinued treatment with aboBoNT-A were assumed to move to the BSC treatment arm (based on clinical practice) and, as such, move between health states following BSC transition probabilities.

The model included cycle lengths of 28 days to capture all relevant changes in resource use, costs, and patient’s health outcome. Additionally, this cycle length was deemed appropriate as it captures long-lasting symptom release and treatment effect as well as matched the length of the treatment cycles in Studies 140 and 142 [19]. Management guidelines published by The Royal College of Physicians indicate that in people with severe and long-standing spasticity, repeated BoNT-A treatments may be required over several years [10]. As a result of this indication, the model was extrapolated for a lifetime horizon. A lifetime horizon allowed the capture of important costs and effects on quality of life (QoL) due to treating patients with aboBoNT-A or BSC.

The model assumes an Australian healthcare payer perspective and resultingly, direct costs and HRQoL outcomes are considered. Both direct costs and patient-specific health outcomes were discounted at a rate of 5% per annum, which is in line with current Pharmaceutical Benefits Advisory Committee (PBAC) submission guidelines [21]. Model outcomes were life years, quality-adjusted life years (QALYs), direct medical costs, and the incremental cost-effectiveness ratio (ICER) presented as cost per QALY gained.

### Data used in the model

#### Modelled population

The modelled population was based on a subgroup of participants enrolled in Studies 140 and 142 (an open label, extension study of Study 140) and consisted of adult patients with lower limb spasticity following a stroke (86.9%) or TBI (13.1%), whose acute event occurred less than 2 years previously [19]. This subgroup was selected from the trials as early treatment initiation demonstrated enhanced functional gains; a correlation was observed highlighting greater improvements in walking speed with shorter time to treating the patient post-event [19]. The average age of the subgroup of Studies 140 and 142 trial participants included in this analysis was 51.7 years and 52.2 years, respectively. In the subgroup population of these studies, 73.9% of patients were receiving concurrent physiotherapy for ALLS and 19.2% were on baclofen, reflective of real-world practice. Patients’ walking speed at baseline varied, with the majority falling into the limited community ambulator category when data from both trials were pooled (Table 1).

**Table 1:** Baseline patient distribution across the health states in the modelled population (n = 84).

Health state	Percentage of patients
Household walker	51.9%
Limited community ambulator	44.4%
Community ambulator	3.7%

#### Clinical inputs

All clinical inputs were derived from clinical trials that are most relevant to the modelled population. For the BSC alone arm, data of patients treated after experiencing an acute event 2 years prior to study randomisation were taken from patients receiving placebo in Study 140, and for the aboBoNT-A plus BSC arm, data of the same subgroup of patients were taken from patients receiving aboBoNT-A in Studies 140 (double-blind) and 142 (open-label extension), maximising the use of available clinical data.

Transition probabilities were calculated based on observed transitions from subgroup individual patient level data from Study 140 and Study 142. Transition probabilities for the two model arms were considered as separate equations in the model due to the trial design and the potential increase in walking speed of patients in the aboBoNT-A arm following treatment. Transition probabilities for aboBoNT-A and BSC were estimated using the combined ‘on treatment’ observations from Study 142 and Study 140, and the placebo arm of Study 140, respectively. These were empirically estimated, and extrapolated beyond the trial period, using a multinomial ordered logit model which determined the probability of a patient transitioning between health states. An ordered multinomial regression was used as it enabled the allocation of cut-off points between the health states. In order to account for the change in various health states over time, time on treatment was incorporated as an independent variable in the model to allow for a gradual reduction in the treatment effect over time. Multiple transformations of the data were considered due to the likeliness of a change in health state of patients occurring. A logarithmic transformation was chosen for the model as it provided the best statistical fit (i.e. p-values ≤0.05) for the transition probabilities for the aboBoNT-A plus BSC arm (Table 2). The logarithmic transformation had the second-best statistical fit for the BSC alone arm and to align the type of transformation for the intervention and the comparator, it was selected for both arms. It was also considered to provide the most clinically viable transition probabilities. To adjust for the small number of patients at later follow-up points, weights were incorporated into the regression analysis using the inverse variance to apply more weight to point estimates that were based on a higher number of observations.

**Table 2:** Transition probabilities (weighted regression estimates) by treatment arm.

	BSC alone				aboBoNT-A plus BSC			
	Coefficient	p-value	95% Confidence Interval		Coefficient	p-value	95% Confidence Interval	
**No transformation on time variable**								
days	–0.0067	0.030	–0.0128	–0.0006	0.0002	0.627	–0.0007	0.0011
/cut1	–2.7996	<0.001	–4.0446	–1.5546	–3.9390	<0.001	–4.5582	–3.3199
/cut2	1.6382	<0.001	0.8003	2.4760	–2.3269	<0.001	–2.7193	–1.9345
/cut3	–	–	–	–	1.9681	<0.001	1.4575	2.4787
/cut4	–	–	–	–	3.1902	<0.001	2.6080	3.7725
**Log transformed time variable**								
ln(days)	–0.3741	0.009	–0.6531	–0.0950	–0.0886	0.053	-0.1782	0.0011
/cut1	–4.3153	<0.001	–5.5854	–3.0452	–4.3430	<0.001	–5.0930	–3.5931
/cut2	0.8771	0.106	–0.1866	1.9407	–2.7091	<0.001	–3.2381	–2.1802
/cut3	–	–	–	–	1.4353	<0.001	0.9912	1.8793
/cut4	–	–	–	–	2.9439	<0.001	2.4746	3.4133

Abbreviations: aboBoNT-A, abobotulinumtoxinA; BSC, best supportive care.

**Fig 2:**
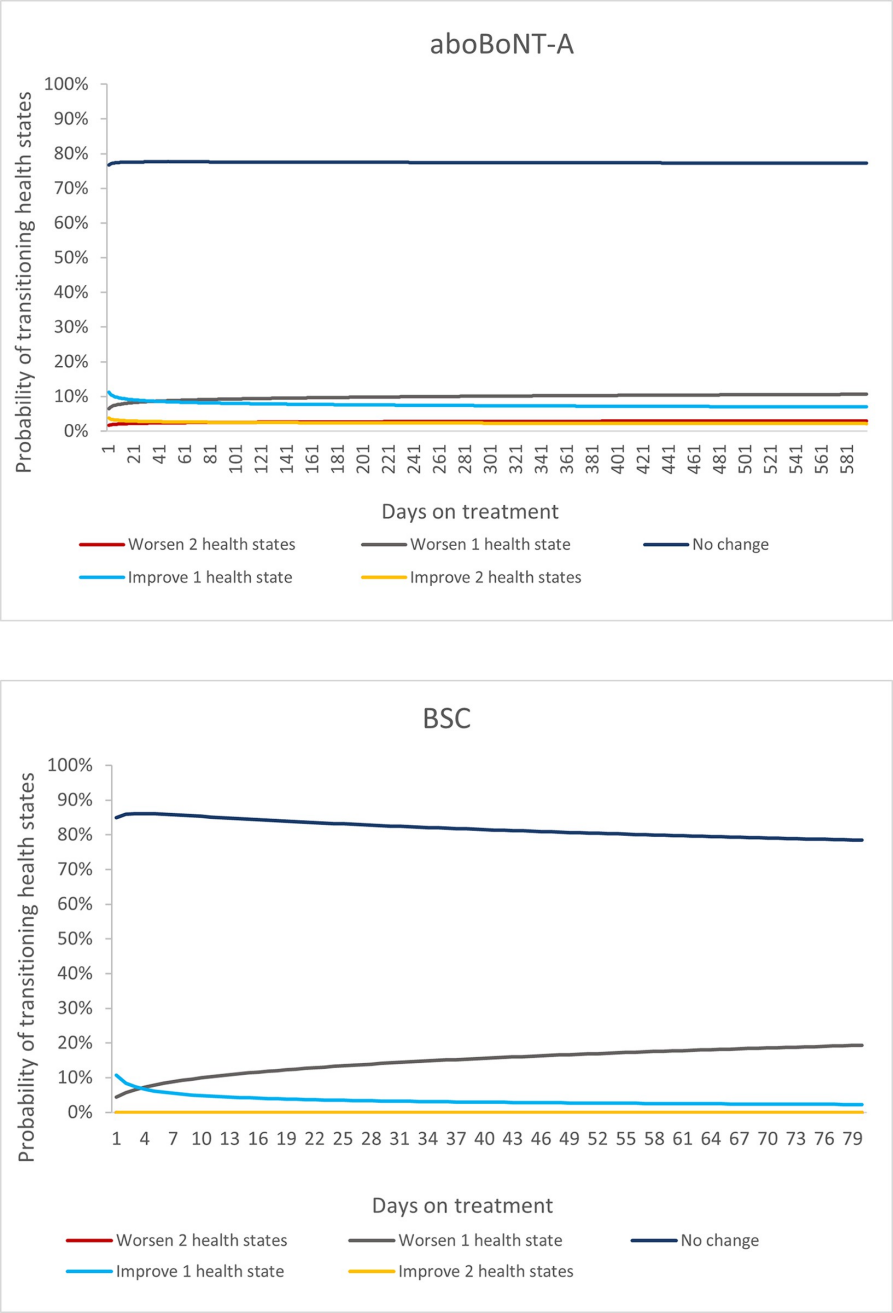
Extrapolation of transition probabilities for patients treated with aboBoNT-A plus BSC and BSC alone over a lifetime horizon. Abbreviations: aboBoNT-A, abobotulinumtoxinA; BSC, best supportive care. Top: Patients treated with aboBoNT-A plus BSC. Bottom: Patients treated with BSC alone. Note: Logarithmic transformation of the time variable in both arms.

Estimates for health state transitions and the time-to-event analysis conducted for treatment discontinuation both include a time dependent variable; this enables extrapolation beyond the observed trial period. Visual representation of the logarithmic transformation of the time variable of these extrapolations is provided in Fig 2. For the base case, to smooth out the transitions between health states, these transformed and extrapolated transition probabilities were used for the entire time horizon, including for the observed trial period.

**Fig 3:**
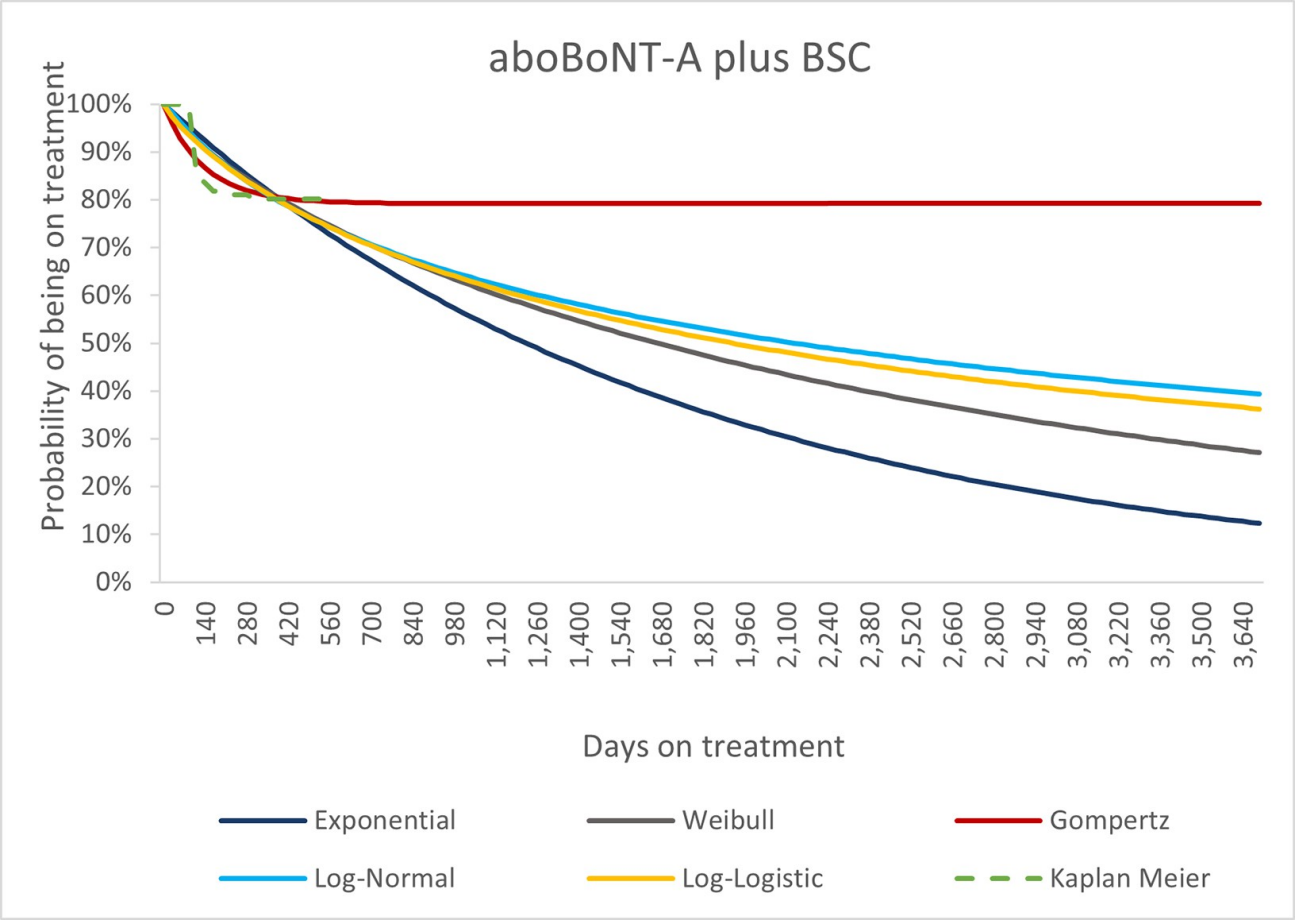
Kaplan Meier curve and fitted parametric curves for permanent study withdrawal. Abbreviations: aboBoNT-A, abobotulinumtoxinA; BSC, best supportive care.

Treatment discontinuation was modelled based on study withdrawals in Study 140 and Study 142 for patients receiving aboBoNT-A and extrapolated beyond the trial period using a Gompertz parametric function (selected based on best statistical fit using the AIC and BIC metrics) as provided in Fig 3. The base case uses this fitted curve for the lifetime horizon to smooth out treatment discontinuation throughout as it would be expected in real world.

An improvement in gait speed of ≥0.13 m/s has been reported as being the minimal clinically important difference in patients with stroke [22]. Therefore, non-responders are defined as patients who did not display a change of ≥0.13 m/s in comfortable barefoot walking speed from baseline in any of their preceding injections or their current injection. In line with Pharmaceutical Benefits Scheme (PBS) criteria, non-responders after four 12-week treatment cycles were required to stop treatment with aboBoNT-A. The proportion of patients who were non-responders and who would have to stop treatment after four cycles of treatment was determined by assessing the individual patient data from Study 142. Based on trial data, it is estimated that 26.2% of patients did not respond to treatment after four treatment cycles. The proportion of patients who had not achieved an improvement in comfortable barefoot walking speed since baseline is provided in Table 3.

**Table 3:** Proportion of non-responders by treatment cycle.

	≤2 year	
	Percentage of non-responders	Number of treatment days
Treatment cycle 1	71.4%	81.3
Treatment cycle 2	50.5%	180.1
Treatment cycle 3	36.7%	274.3
Treatment cycle 4	26.2%	370.1

As ALLS doesn’t impact mortality, background mortality was included based on the two main underlying conditions: stroke [23] and TBI [24]. The mortality associated with these two events was adjusted by age and gender, aligning with the aboBoNT-A clinical trial population [19].

#### Utility, resource use and cost data

Specific HRQoL measurements collected in Studies 140 and 142 using the 5-level European Quality of Life 5-Dimension (EQ-5D-5L) tool were converted to Australian QoL estimates using utility weights based on a discrete choice experiment conducted by the Cancer Research Economics Support Team, University of Technology Sydney (CREST UTS) [25].

Drug acquisition, administration and resource use costs for aboBoNT-A were considered in the model. In the base case analysis resource use accounted for neurologist, physiotherapist, and primary care physician visits as well as patients requiring a splint and days spent in hospitalisation [26–28]. Costs associated with a reduction in the volume of oral anti-spasmodic treatments (and associated costs relating to adverse events) and any costs following surgical procedures have been excluded from the analysis due to lack of resource use and cost data available specific to this therapy area in the Australian setting to minimise uncertainty in the base case analysis. The acquisition cost of aboBoNT-A was obtained from the Pharmaceutical Benefits Scheme’s Dispensed Price for Maximum Quantity (PBS DPMQ) [29], and the administration cost was the fee associated with the treatment obtained from the Medicare Benefits Schedule (MBS) [30]. Information for the estimation of the number of vials of aboBoNT-A required per treatment cycle was sourced from Studies 140 and 142 [19].

According to the study protocols for Studies 140 and 142, patients were eligible to receive aboBoNT-A with an interval of a minimum of 12-weeks between cycles [19]. However, the duration (weeks ± SD) of the observed interval varied between cycles ranging from 10.1 ± 4.3 weeks to 15.3 ± 5.2 weeks. This variation was accounted for in the model by estimating the probability of retreatment per model cycle based on the mean number of treatments per patient (3.855) and the mean study duration (378.835 days).

All costs are presented in 2020 Australian dollars (AUD). The resource use, cost and utility input values applied in the model for the base case are provided in Table 4.

**Table 4:** Model inputs.

Parameter	Input		PSA distribution	Source
Non-responders after four treatment cycles (370.1 days)	26.2%		N/A	Studies 140 and 142
Retreatment rate, per model cycle	0.01		Beta	Studies 140 and 142
**Utilities**				
Household walker	0.4048		Beta	Studies 140 and 142; CREST UTS
Limited community ambulator	0.4918		Beta	Studies 140 and 142; CREST UTS
Community ambulator	0.5400		Beta	Studies 140 and 142; CREST UTS
**Resource use per patient**				
	aboBoNT-A plus BSC	BSC		
Neurologist visit (per year)	4.00	1.90	Gamma	Ward et al., 2005 [31]^†^
Physiotherapist visit (per year)	3.69	3.48	Gamma	Shackley et al., 2012 [26]^‡^
Primary care physician visit (per year)	0.85	0.59	Gamma	Shackley et al., 2012 [26]^‡^
Days of hospitalisation (per year)	0.23	0.83	Gamma	Shackley et al., 2012 [26]^‡^
Splint (per year)	0.23	0.29	Gamma	Shaw et al., 2010 [27]
Average number of vials (per treatment cycle)	2.66	0	Gamma	Studies 140 and 142
**Costs**				
500 IU vial	$523.75		Gamma^§^	PBS DPMQ [29]
Administration	$124.95		Gamma	MBS [30]
Neurologist visit	$134.70		Gamma	MBS [30]
Physiotherapist visit	$65.85		Gamma	MBS [30]
Primary care physician visit	$95.13		Gamma	MBS [30]
Hospitalisation (per day)	5,892.00		Gamma	NWAU calculator 2022 [32]
Splint	$59.70		Gamma	MBS [30]

Abbreviations: AUD, Australian dollar; CREST UTS, Cancer Research Economics Support Team, University of Technology Sydney; MBS, Medicare Benefits Schedule; N/A, not applicable; PBS DPMQ, Pharmaceutical Benefits Scheme’s Dispensed Price for Maximum Quantity; PSA, probabilistic sensitivity analysis.

† The number of neurologist visits as mentioned in the publication.

‡ This figure has been calculated from the mean number of contacts in relation to the complete study population as mentioned in the publication.

§ In line with standard practice, cost of the vial is not included in the PSA as the price is fixed and there is no uncertainty in the value.

Note: Costs are in AUD.

#### Sensitivity analyses

Several scenario analyses and a probabilistic sensitivity analysis (PSA) were conducted to explore the uncertainty in the model parameters used in the base case analysis. In the PSA, distributions were assigned to the model parameters as per standard practice; distributions applied are presented in Table 4. For the treatment discontinuation curves and the transition probabilities, Cholesky decomposition matrices were used in the PSA. To generate the PSA results, the model was run over 1,000 simulations. In the additional scenario analyses, inputs and assumptions around transition probabilities, treatment discontinuation, stopping rules, utility values, resource use and costs, discount rate, and time horizons were tested. The list of inputs used for the base case and sensitivity analyses can be found in the supplementary materials (S1 Table) along with the scenario analyses values (S2 Table).

## Results

### Base case

Analysis of the base case indicates that aboBoNT-A plus BSC is cost-effective versus BSC alone, yielding an ICER of $35,721 per QALY gained. Base case results are provided in Table 5. The ICER is below the commonly acceptable willingness-to-pay (WTP) threshold of $75,000 per QALY for cost-effectiveness used in Australia. A WTP of $75,000 has previously been used in PBAC submissions in renal cell carcinoma [33], follicular lymphoma [34], and intraocular lens technology [35].

**Table 5:** Base case results.

	Costs				Total LYs	Total QALYs	Cost per QALY ($)
	Acquisition	Administration	Other costs	Total			
AboBoNT-A plus BSC	21,759	1,952	30,277	53,988	8.36	3.78	–
BSC	0	0	45,526	45,526	8.36	3.54	–
**Incremental**	**21,759**	**1,952**	**–15,248**	**8,462**	**0.00**	**0.24**	**35,721**

Abbreviations: AUD, Australian dollar; BSC, best supportive care; LY, life year; QALY, quality adjusted life year.

Note: All values are discounted at 5% per annum. Costs are in AUD.

### Sensitivity analyses

The results from the sensitivity analyses for aboBoNT-A plus BSC versus BSC alone indicate that the base case results are largely within a cost-effective range of less than $75,000 per QALY gained. While the base case analysis presents a conservative estimate, adjusting individual model parameters produces changes to the ICER for each analysis performed. Inputs and assumptions that produced higher ICERs include the use of an alternative HRU utilisation source, the use of unweighted regression analyses to determine regression probabilities, declining to implement a stopping rule, applying shorter time horizons, incorporating upper bound estimates for utilities, and all transitions are based on Study 140. Results from the sensitivity analyses are provided in Table 6.

**Table 6:** Scenario analyses for alternative model inputs and assumptions.

	Incremental costs	Incremental QALYs	ICER (cost per QALY)
**Base case**	**$8,462**	**0.24**	**$35,721**
**Transition probabilities**			
Both arms: No transformation of time	$8,462	0.44	$19,025
aboBoNT-A plus BSC arm: No transformation of time	$8,462	0.38	$21,992
BSC arm: No transformation of time	$8,462	0.30	$28,483
Use observed data for the trial period followed by extrapolated curves	$10,920	0.40	$27,341
Unweighted regression estimates	$8,462	0.17	$49,312
All transitions based on Study 140	$8,462	0.16	$52,018
**Distribution of the permanent treatment discontinuation curves**			
Exponential	$6,863	0.33	$20,600
Weibull	$5,502	0.20	$27,177
Log-Normal	$4,764	0.12	$39,437
Log-Logistic	$4,678	0.12	$37,889
Kaplan Meier, with constant tail	$8,537	0.24	$35,488
**Stopping rule**			
For non-responders at treatment cycle 1	$1,958	0.04	$49,783
For non-responders at treatment cycle 2	$5,022	0.13	$38,136
For non-responders at treatment cycle 3	$7,003	0.19	$36,483
No stopping rule	$11,779	0.34	$34,443
**Utility values**			
Upper 95% CI bound	$8,462	0.18	$46,280
Lower 95% CI bound	$8,462	0.29	$29,094
**Resource utilisation**			
Three vials per injection	$11,244	0.24	$47,466
Two vials per injection	$3,064	0.24	$12,933
Including HCRU costs associated with general disease management for 2 years following treatment initiation for both arms	$19,577	0.24	$82,642
Including HCRU costs associated with general disease management for 5 years following treatment initiation for both arms	$16,236	0.24	$68,538
Alternative resource use source of Danchenko et al. 2022 [36]. Limited community ambulator classified as responder	$24,180	0.24	$102,075
Alternative resource use source of Danchenko et al. 2022 [36]. Limited community ambulator classified as non-responder	$23,990	0.24	$101,272
**Time horizon**			
2 years	$2,615	0.03	$77,514
5 years	$4,373	0.09	$47,580
10 years	$6,212	0.16	$39,434
**Discount rate for both costs and outcomes**			
0%	$13,272	0.40	$32,860
3.5%	$9,463	0.27	$34,834

Abbreviations: aboBoNT-A, abobotulinumtoxinA; BSC, best supportive care; CI, confidence interval; HCRU; healthcare resource utilisation; ICER, incremental cost-effectiveness ratio; QALY, quality adjusted life year.

Note: Costs are in AUD.

**Fig 4:**
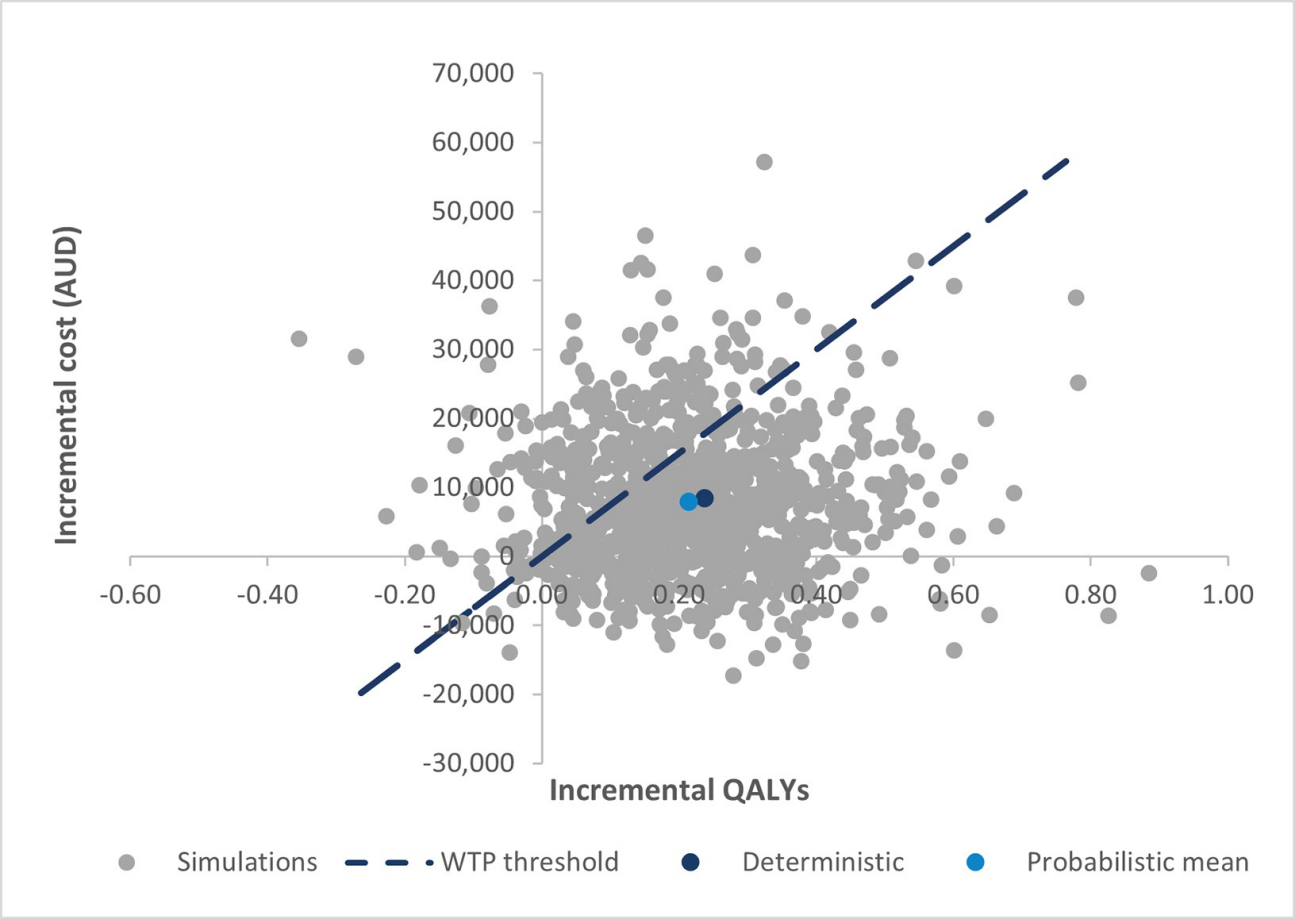
PSA cost-effectiveness plane. Abbreviations: AUD, Australian dollar; PSA, probabilistic sensitivity analysis; QALY, quality adjusted life year; WTP, willingness to pay.

**Fig 5:**
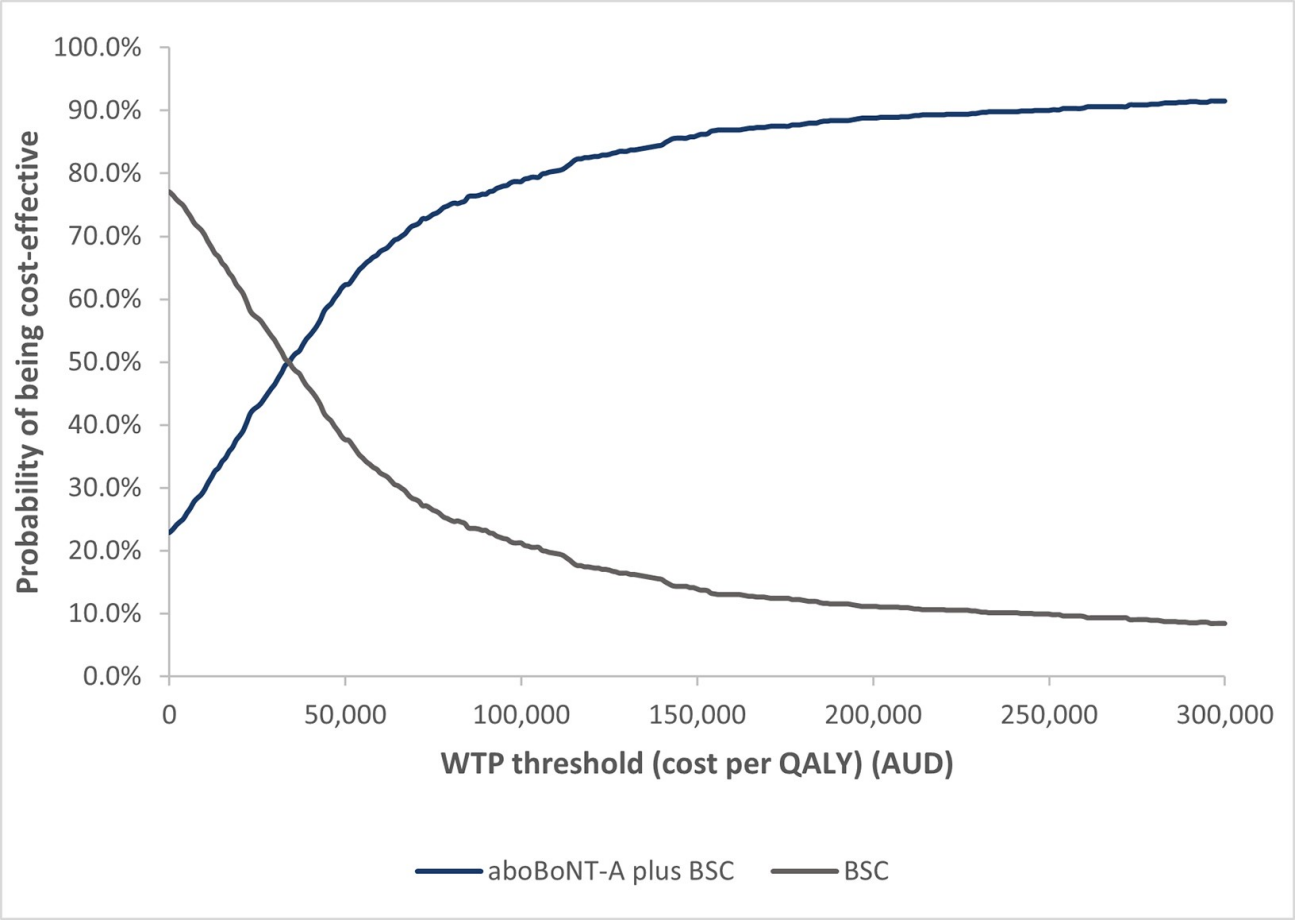
Cost-effectiveness acceptability curve. Abbreviations: AUD, Australian dollar; BSC, best supportive care; QALY, quality adjusted life year; WTP, willingness to pay.

The scatterplot of the simulations performed in the PSA and the cost-effectiveness acceptability curve (CEAC) are presented in Figs 4 and 5, respectively. The majority of iterations in the scatterplot fall inside the north-east quadrant, indicating a treatment that is both more costly and more effective than BSC alone. The CEAC results show that aboBoNT-A plus BSC has a 74% probability of being cost-effective compared to BSC alone at a WTP threshold of $75,000 per QALY. The PSA outcomes confirm that the base case results are fairly robust and that aboBoNT-A plus BSC is a cost-effective treatment compared to BSC alone.

## Discussion

Results from this cost-utility analysis of aboBoNT-A plus BSC versus BSC alone indicate that aboBoNT-A plus BSC is a cost-effective treatment from the Australian payer perspective as the ICER observed in the base case is below the commonly acceptable WTP threshold of $75,000 per QALY applied in Australia. Furthermore, varying treatment parameters in sensitivity analyses yielded ICERs that are also largely within this cost-effectiveness threshold in Australia, thereby validating the robustness of the base case analysis. Higher ICERs (the majority of which were still below the WTP threshold) were observed when healthcare resource use data by Danchenko et al. was used, when shorter time horizons were applied, when HRU use was applied for a short time rather than for the lifetime horizon, when unweighted regression analyses were used to determine transition probabilities, and when no stopping rule was applied to treatment non-responders.

The alternative resource use source applied in the scenario analysis examined resource use for managing spasticity and cervical dystonia [36]. The study by Danchenko et al., 2022, gathered resource use data for managing adult upper limb spasticity via survey; lower limb spasticity was not included. Therefore, the study assumed that the resource use of lower limb spasticity is equal to that of upper limb. Implementing this resource use source increased the ICER in the model. This increase can be attributed to the similarity between responder and non-responder health states. The source provides resource use statistics for 14 individual resources, but a small difference is only reported between health states in four of the resources (general practitioner visits, specialist visits, pain clinic visits, and occupational therapy).

Applying HRU costs for a shorter time horizon increases the ICER considerably due to the loss in potential savings caused by an important reduction in HRU costs that aboBoNT-A patients incur throughout their lifetime compared to BSC patients.

Applying a shorter time horizon increases the ICER considerably due to an upfront cost of treatment with aboBoNT-A applied for all patients, which later decreases as some patients stop receiving the treatment. In addition, differences in accumulated QALYs between the two model arms start to arise from around year 2.

A higher ICER was observed in the use of unweighted regression analysis to determine regression probabilities. This can be attributed to unweighted regression analyses having a greater potential to be influenced by estimates that have a higher level of uncertainty due to a lower number of patients contributing to the estimates in the latter stages of the observation period.

A reduction in walking performance of an individual can create a social disadvantage and can be indicative of a decline in general health state and predictive of future health concerns [20]. A stopping rule for patients not responding to treatment has been implemented in this analysis as patients who have not responded within the first four treatment cycles are most likely to be non-responders for subsequent treatment cycles [19]. A higher ICER is observed when no stopping rule is applied, and patients continue to receive treatment regardless of their response. Ample opportunity exists for the patient to respond to the treatment by cycle 4 of the treatment, and this is corroborated by the observation that excluding patients who are non-responders at treatment cycle 1 results in a substantially lower ICER. The stopping rule was included in sensitivity analyses because of data related to the response of patients to treatment observed in pivotal clinical trials [19]. The stopping rule was based on the patient responding to treatment by achieving a minimal clinically important improvement in comfortable barefoot walking speed of ≥0.13 m/s over four treatment cycles. The proportion of non-responders in the population declines as the total number of treatment cycles increases (Table 3) thereby validating the efficacy of treatment with aboBoNT-A. This is in line with reports of patients receiving early BoNT-A intervention closer to the time of the acute event having better clinical outcomes [17, 37].

Results in the PSA may suggest moderate robustness of the base case results which is likely to be caused by the higher level of uncertainty in the underlying clinical data used for the BSC only arm. For this model arm, only one treatment cycle was available from the placebo arm of Study 140 as patients were switched to active treatment as part of the open-label extension study following the first blinded treatment cycle.

Limitations to the analysis include the fact that healthcare resource use, both in the base case and scenario analysis, relate to adult upper limb spasticity and are assumed to apply for lower limb spasticity in the same way. In addition, resource use costs, such as costs associated with anti-spasmodic treatments and their adverse effects, were not incorporated into the analysis particularly for best standard of care comparisons. This may result in the ICER being overestimated, and thus it is expected that including these additional costs would enhance the cost-effectiveness of aboBoNT-A plus BSC. Botox exclusion as a comparator is also considered a limitation. However, as previously mentioned, published studies seldom report walking speed, an ITC could not be conducted.

A further limitation of the analysis is that transitions are not observed at continuous timepoints. Instead, the information is in the form of panel data such that the transition between two states is observed only at the end of each 12-week cycle. Given that the data are observed at fixed timepoints, and individuals are not followed continually over time, the temporal aspect of transitions between states, which multi-state models capture, are not relevant. Therefore, we concluded that it was sufficient to use an ordered multinomial model and predict the probabilities of the health state depending on the time on treatment (through the dependence on ln[days]) as opposed to a multistate model in which the transition probabilities are inherently time dependent.

The conclusion from the present analysis, indicating that treatment with BoNT-A, while more costly, is a cost-effective measure in the treatment of post-stroke or TBI spasticity, is corroborated by published evidence. Clinical trials of aboBoNT-A have demonstrated a reduction in muscle tone in the lower limb versus placebo when administered to patients with ALLS [19]. The reduction in muscle tone alongside the longer duration of symptom relief, and the improved functional gains resulting from early initiation of aboBoNT-A treatment provide clinical benefits to this population [19]. While there is a paucity of information on cost-effectiveness analyses of BoNT-A treatment in patients with ALLS, cost-effectiveness analyses of BoNT-A in patients with other forms of post-stroke or TBI spasticity have reported BoNT-A being a cost-effective form of treatment versus BSC [31, 38–41]. Management of post-stroke or TBI spasticity with BoNT-A as an adjunct to rehabilitation has been reported as being cost-effective versus rehabilitation alone [39–41]. The cost-effectiveness of BoNT-A compared with oral therapy for post-stroke or TBI spasticity has also been reported highlighting the benefits of using this form of treatment in first- and second-line therapy [31]. These publications indicate that an improvement in disability is associated with BoNT-A treatment, which translates into greater QALYs. However, it must be noted that across these studies, treatment with BoNT-A is associated with higher treatment costs than BSC [31, 38–41].

A clinical and pharmacoeconomic benefit to patients and healthcare systems is associated with early treatment of spasticity. The ONTIME study investigated the progression of upper limb spasticity following stroke [17]. An optimal time for management of the condition post-stroke was identified and early treatment with aboBoNT-A was recommended. The present study corroborates evidence that early intervention for post-stroke management results in optimal outcomes for patients. The choice of the trial population for this cost-effectiveness analysis was based on the premise that it is representative of patients in Australia who would be eligible for aboBoNT-A and treated early for ALLS as standard clinical practice changes, and therefore, the findings are generalisable to the Australian practice.

Results from clinical trials have demonstrated the efficacy of aboBoNT-A in patients with ALLS [19], and results from this study demonstrate the cost-effectiveness of using this treatment in this population. International and Australian treatment guidelines include similar recommendations relating to how spasticity should be managed and have similar indications on the clinical benefit of BoNT-A in treating patients with lower limb spasticity [10–14]. The efficacy results versus placebo in clinical trials, coupled with the cost-effectiveness reported in this publication, specifically for patients with ALLS treated within 2 years of an acute event, indicate that aboBoNT-A plus BSC is a better value for money and more efficient treatment choice than BSC alone in Australia.

## Supporting information

S1 Table Parameters used for the base case and the sensitivity analyses. Abbreviations: aboBoNT-A, abobotulinumtoxinA; AUD, Australian dollar; BSC, best supportive care; PBS DPMQ, Pharmaceutical Benefits Scheme’s Dispensed Price for Maximum Quantity; MBS, Medicare Benefits Schedule; TP, transition probability; QoL, quality of life. *In line with standard practice, cost of the vial is not included in the PSA as the price is fixed and there is no uncertainty in the value.
(DOCX)

S2 Table Parameters used for the scenario analyses. Abbreviations: aboBoNT-A, abobotulinumtoxinA; AUD, Australian dollar; BSC, best supportive care; MBS, Medicare Benefits Schedule; PBS DPMQ, Pharmaceutical Benefits Scheme’s Dispensed Price for Maximum Quantity; TP, transition probability.
(DOCX)
