# Supplementary material for: Cost-effectiveness of abobotulinumtoxinA plus best supportive care compared with best supportive care alone for early treatment of adult lower limb spasticity following an acute event
Source: PLoS One. 2024 Feb 1;19(2):e0296340. doi: 10.1371/journal.pone.0296340 (PMC10833516; doi:10.1371/journal.pone.0296340)
Supplement: S1 Table — Abbreviations: aboBoNT-A, abobotulinumtoxinA; AUD, Australian dollar; BSC, best supportive care; PBS DPMQ, Pharmaceutical Benefits Scheme’s Dispensed Price for Maximum Quantity; MBS, Medicare Benefits Schedule; TP, transition probability; QoL, quality of life. *In line with standard practice, cost of the vial is not included in the PSA as the price is fixed and there is no uncertainty in the value. (DOCX) [file pone.0296340.s001.docx]

S1 Table

Electronic Supplementary Material

Cost-effectiveness of abobotulinumtoxinA plus best supportive care compared to best supportive care alone for early treatment of adult lower limb spasticity following an acute event

**S1 Table: Parameters used for the base case and the sensitivity analyses**

**Authors:**

Peter Moore^1^, Natalya Danchenko^2^, Diana Weidlich^3^, Alejandra Rodarte Tijerina^3^

^1^ Ipsen, Melbourne, VIC, Australia;

^2^ Ipsen Global, Boulogne-Billancourt, France;

^3^ Clarivate, London, UK

**Corresponding Author:**

Peter Moore, [ptmoorie@hotmail.com](mailto:ptmoorie@hotmail.com)

S1 Table: Parameters used for the base case and the sensitivity analyses

| **Parameter** | **Base case** | **Standard error** | **Lower 95% CI** | **Upper 95% CI** | **Distribution** | **Source** |
| --- | --- | --- | --- | --- | --- | --- |
| Discount rate for costs and outcomes | 5% | – | – | – | – | PBAC [1] |
| Proportion of stroke patients | 0.869 | 0.1738 | 0.53 | 1.00 | Beta | Studies 140 and 142 [2] |
| Proportion of traumatic brain injury patients | 0.131 | 0.0262 | 0.08 | 0.18 | Beta | Studies 140 and 142 [2] |
| Post stroke mortality year 1 | 0.084 | 0.01678 | 0.05 | 0.12 | Beta | Peng et al., 2022 [3] |
| Post stroke mortality year 2 | 0.081 | 0.01624 | 0.05 | 0.11 | Beta | Peng et al., 2022 [3] |
| Post stroke mortality year 3 | 0.080 | 0.01598 | 0.05 | 0.11 | Beta | Peng et al., 2022 [3] |
| Post stroke mortality year 4 | 0.080 | 0.01598 | 0.05 | 0.11 | Beta | Peng et al., 2022 [3] |
| Post stroke mortality year 5 | 0.077 | 0.01534 | 0.05 | 0.11 | Beta | Peng et al., 2022 [3] |
| Post stroke mortality year 5+ | 0.077 | 0.01534 | 0.05 | 0.11 | Beta | Peng et al., 2022 [3] |
| Mortality after TB1 age group 16-39 year 0 | 0.009 | 0.001855 | 0.01 | 0.01 | Beta | Fuller et al., 2016 [4] |
| Mortality after TB1 age group 16-39 year 5 | 0.019 | 0.003711 | 0.01 | 0.03 | Beta | Fuller et al., 2016 [4] |
| Mortality after TB1 age group 16-39 year 10 | 0.022 | 0.004453 | 0.01 | 0.03 | Beta | Fuller et al., 2016 [4] |
| Mortality after TB1 age group 16-39 year 15 | 0.033 | 0.006679 | 0.02 | 0.05 | Beta | Fuller et al., 2016 [4] |
| Mortality after TB1 age group 16-39 year 20 | 0.033 | 0.006679 | 0.02 | 0.05 | Beta | Fuller et al., 2016 [4] |
| Mortality after TB1 age group 16-39 year 25 | 0.033 | 0.006679 | 0.02 | 0.05 | Beta | Fuller et al., 2016 [4] |
| Mortality after TB1 age group 40-64 year 0 | 0.036 | 0.007194 | 0.02 | 0.05 | Beta | Fuller et al., 2016 [4] |
| Mortality after TB1 age group 40-64 year 5 | 0.079 | 0.015827 | 0.05 | 0.11 | Beta | Fuller et al., 2016 [4] |
| Mortality after TB1 age group 40-64 year 10 | 0.108 | 0.021583 | 0.07 | 0.15 | Beta | Fuller et al., 2016 [4] |
| Mortality after TB1 age group 40-64 year 15 | 0.151 | 0.030216 | 0.09 | 0.21 | Beta | Fuller et al., 2016 [4] |
| Mortality after TB1 age group 40-64 year 20 | 0.187 | 0.03741 | 0.11 | 0.26 | Beta | Fuller et al., 2016 [4] |
| Mortality after TB1 age group 40-64 year 25 | 0.187 | 0.03741 | 0.11 | 0.26 | Beta | Fuller et al., 2016 [4] |
| Mortality after TB1 age group >65 years year 0 | 0.275 | 0.054945 | 0.17 | 0.38 | Beta | Fuller et al., 2016 [4] |
| Mortality after TB1 age group >65 years year 5 | 0.505 | 0.101099 | 0.31 | 0.70 | Beta | Fuller et al., 2016 [4] |
| Mortality after TB1 age group >65 years year 10 | 0.714 | 0.142857 | 0.43 | 0.99 | Beta | Fuller et al., 2016 [4] |
| Mortality after TB1 age group >65 years year 15 | 0.813 | 0.162637 | 0.49 | 1.00 | Beta | Fuller et al., 2016 [4] |
| Mortality after TB1 age group >65 years year 20 | 0.835 | 0.167033 | 0.51 | 1.00 | Beta | Fuller et al., 2016 [4] |
| Mortality after TB1 age group >65 years year 25 | 0.835 | 0.167033 | 0.51 | 1.00 | Beta | Fuller et al., 2016 [4] |
| Treatment discontinuation: Gompertz - lambda | –6.446 | – | – | – | Cholesky decomposition | Studies 140 and 142 [2] |
| Treatment discontinuation: Gompertz - kappa | –0.007 | – | – | – | Cholesky decomposition | Studies 140 and 142 [2] |
| TP - aboBoNT­A plus BSC - days coefficient (log transformed) | –0.0886 | 0.0457 | –0.1782 | 0.0011 | Cholesky decomposition | Studies 140 and 142 [2] |
| TP - aboBoNT­A plus BSC - /cut1 coefficient (log transformed) | –4.3430 | 0.3826 | –5.0930 | –3.5931 | Cholesky decomposition | Studies 140 and 142 [2] |
| TP - aboBoNT­A plus BSC - /cut2 coefficient (log transformed) | –2.7091 | 0.2699 | –3.2381 | –2.1802 | Cholesky decomposition | Studies 140 and 142 [2] |
| TP - aboBoNT­A plus BSC - /cut3 coefficient (log transformed) | 1.4353 | 0.2266 | 0.9912 | 1.8793 | Cholesky decomposition | Studies 140 and 142 [2] |
| TP - aboBoNT­A plus BSC - /cut4 coefficient (log transformed) | 2.9439 | 0.2395 | 2.4746 | 3.4133 | Cholesky decomposition | Studies 140 and 142 [2] |
| TP - BSC - days coefficient (log transformed) | –0.3741 | 0.1424 | –0.6531 | –0.0950 | Cholesky decomposition | Study 140 [2] |
| TP - BSC - /cut1 coefficient (log transformed) | - | 0.0000 | 0.0000 | 0.0000 | Cholesky decomposition | Study 140 [2] |
| TP - BSC - /cut2 coefficient (log transformed) | –4.3153 | 0.6480 | –5.5854 | –3.0452 | Cholesky decomposition | Study 140 [2] |
| TP - BSC - /cut3 coefficient (log transformed) | 0.8771 | 0.5427 | –0.1866 | 1.9407 | Cholesky decomposition | Study 140 [2] |
| TP - BSC - /cut4 coefficient (log transformed) | – | 0.0000 | 0.0000 | 0.0000 | Cholesky decomposition | Study 140 [2] |
| Baseline health state distribution – Household walker | 51.90% | – | 0.42 | 0.62 | Dirichlet | Studies 140 and 142 [2] |
| Baseline health state distribution - Limited community ambulator | 44.40% | – | 0.36 | 0.53 | Dirichlet | Studies 140 and 142 [2] |
| Baseline health state distribution - Community ambulator | 3.70% | – | 0.03 | 0.04 | Dirichlet | Studies 140 and 142 [2] |
| Proportion of non-responders at treatment cycle 4 | 26.2% | 0.0226 | 0.22 | 0.31 | Beta | Studies 140 and 142 [2] |
| Rate of retreatment per day | 0.01 | 0.0051 | 0.00 | 0.02 | Beta | Studies 140 and 142 [2] |
| Cost of vial (500 U) (AUD) | 523.75 | 53.4449 | 419.00 | 628.50 | Gamma* | PBS DPMQ [5] |
| Average number of vials per treatment cycle | 2.66 | 0.2714 | 2.13 | 3.19 | Gamma | Studies 140 and 142 [2] |
| Cost of administration (AUD) | 124.95 | 12.7502 | 99.96 | 149.94 | Gamma | MBS [6] |
| Neurologist visit - number per year per patient - aboBoNT­A plus BSC | 4.00 | 0.4082 | 3.20 | 4.80 | Gamma | Ward et al., 2014 [7] |
| Neurologist visit - number per year per patient - BSC | 1.90 | 0.1939 | 1.52 | 2.28 | Gamma | Ward et al., 2014 [7] |
| Neurologist visit - cost per visit (AUD) | 134.70 | 13.7452 | 107.76 | 161.64 | Gamma | PBS DPMQ [5] |
| Physiotherapist visit - number per year per patient - aboBoNT­A plus BSC | 3.69 | 0.3765 | 2.95 | 4.43 | Gamma | Shackley et al., 2012 [8] |
| Physiotherapist visit - number per year per patient - BSC | 3.48 | 0.3551 | 2.78 | 4.18 | Gamma | Shackley et al., 2012 [8] |
| Physiotherapist visit - cost per visit (AUD) | 65.85 | 6.7185 | 52.68 | 79.02 | Gamma | MBS [6] |
| Primary care physician visit - number per year per patient - aboBoNT­A plus BSC | 0.85 | 0.0867 | 0.68 | 1.02 | Gamma | Shackley et al., 2012 [8] |
| Primary care physician visit - number per year per patient - BSC | 0.59 | 0.0602 | 0.47 | 0.71 | Gamma | Shackley et al., 2012 [8] |
| Primary care physician visit - cost per visit (AUD) | 95.13 | 9.7073 | 76.10 | 114.16 | Gamma | MBS [6] |
| Days of hospitalisation - number per year per patient - aboBoNT­A plus BSC | 0.23 | 0.0235 | 0.18 | 0.28 | Gamma | Shackley et al., 2012 [8] |
| Days of hospitalisation - number per year per patient - BSC | 0.83 | 0.0847 | 0.66 | 1.00 | Gamma | Shackley et al., 2012 [8] |
| Days of hospitalisation - cost per day (AUD) | 5,892 | 601.2355 | 4713.60 | 7070.40 | Gamma | AR-DRG [9] |
| Splint - number per year per patient - aboBoNT­A plus BSC | 0.23 | 0.0235 | 0.18 | 0.28 | Gamma | Shaw et al., 2010 [10] |
| Splint - number per year per patient - BSC | 0.29 | 0.0296 | 0.23 | 0.35 | Gamma | Shaw et al., 2010 [10] |
| Splint - cost per unit (AUD) | 59.70 | 6.0919 | 47.76 | 71.64 | Gamma | MBS [6] |
| QoL - Household walker regression utility | 0.4048 | 0.0396 | 0.33 | 0.48 | Beta | Studies 140 and 142 [2] |
| QoL - Limited community ambulator regression utility | 0.4918 | 0.0355 | 0.42 | 0.56 | Beta | Studies 140 and 142 [2] |
| QoL - Community ambulator regression utility | 0.5400 | 0.0197 | 0.50 | 0.58 | Beta | Studies 140 and 142 [2] |

Abbreviations: aboBoNT­A, abobotulinumtoxinA; AUD, Australian dollar; BSC, best supportive care; PBS DPMQ, Pharmaceutical Benefits Scheme’s Dispensed Price for Maximum Quantity; MBS, Medicare Benefits Schedule; TP, transition probability; QoL, quality of life.

*In line with standard practice, cost of the vial is not included in the PSA as the price is fixed and there is no uncertainty in the value.

References

1. The Pharmaceutical Benefits Advisory Committee Guidelines. Guidelines for preparing submissions to the Pharmaceutical Benefits Advisory Committee (PBAC) Version 5.0. Available at https://pbac.pbs.gov.au/. Accessed 7th May, 2021. 2016.

2. Gracies JM, Esquenazi A, Brashear A, Banach M, Kocer S, Jech R, et al. Efficacy and safety of abobotulinumtoxinA in spastic lower limb: Randomized trial and extension. Neurology. 2017 Nov 28;89[2]2):2245-53.

3. Peng Y, Ngo L, Hay K, Alghamry A, Colebourne K, Ranasinghe I. Long-Term Survival, Stroke Recurrence, and Life Expectancy After an Acute Stroke in Australia and New Zealand From 2008-2017: A Population-Wide Cohort Study. Stroke. 2022 Aug;53(8):2538-48.

4. Fuller GW, Ransom J, Mandrekar J, Brown AW. Long-Term Survival Following Traumatic Brain Injury: A Population-Based Parametric Survival Analysis. Neuroepidemiology. 2016;47[1]:1-10.

5. Australian Government, Department of Health.,. Pharmaceutical Benefits Scheme Costs: Schedule of Pharmaceutical Benefits (Volume 2 – Section 100), 1 July 2020. Available at https://www.pbs.gov.au/publication/schedule/2020/07/2020-07-01-general-schedule-volume-2.pdf. Accessed 16 July 2021. 2020.

6. Australian Government, Department of Health. MBS costs: Medicare Benefits Schedule, July 2020. Available at http://www.mbsonline.gov.au/internet/mbsonline/publishing.nsf/Content/Home. 2020.

7. Ward AB, Wissel J, Borg J, Ertzgaard P, Herrmann C, Kulkarni J, et al. Functional goal achievement in post-stroke spasticity patients: the BOTOX® Economic Spasticity Trial (BEST). J Rehabil Med. 2014 Jun;46(6):504-13.

8. Shackley P, Shaw L, Price C, van Wijck F, Barnes M, Graham L, et al. Cost-effectiveness of treating upper limb spasticity due to stroke with botulinum toxin type A: results from the botulinum toxin for the upper limb after stroke (BoTULS) trial. Toxins (Basel). 2012 Dec;4(12):1415-26.

9. Australian Government, Australian Institute of Health and Welfare. AR-DRG: Australian Refined – Diagnostic Related Groups, Public Hospital casemix costs (Round 16).

10. Shaw L, Rodgers H, Price C, van Wijck F, Shackley P, Steen N, et al. BoTULS: a multicentre randomised controlled trial to evaluate the clinical effectiveness and cost-effectiveness of treating upper limb spasticity due to stroke with botulinum toxin type A. Health Technol Assess. 2010 May;14[2]6):1-113, iii-iv.
