# Supplementary material for: Cost-effectiveness of abobotulinumtoxinA plus best supportive care compared with best supportive care alone for early treatment of adult lower limb spasticity following an acute event
Source: PLoS One. 2024 Feb 1;19(2):e0296340. doi: 10.1371/journal.pone.0296340 (PMC10833516; doi:10.1371/journal.pone.0296340)
Supplement: S2 Table — Abbreviations: aboBoNT-A, abobotulinumtoxinA; AUD, Australian dollar; BSC, best supportive care; MBS, Medicare Benefits Schedule; PBS DPMQ, Pharmaceutical Benefits Scheme’s Dispensed Price for Maximum Quantity; TP, transition probability. (DOCX) [file pone.0296340.s002.docx]

S2 Table

Electronic Supplementary Material

Cost-effectiveness of abobotulinumtoxinA plus best supportive care compared to best supportive care alone for early treatment of adult lower limb spasticity following an acute event

S2 Table: Parameters used for the scenario analyses

**Authors:**

Peter Moore^1^, Natalya Danchenko^2^, Diana Weidlich^3^, Alejandra Rodarte Tijerina^3^

^1^ Ipsen, Melbourne, VIC, Australia;

^2^ Ipsen Global, Boulogne-Billancourt, France;

^3^ Clarivate, London, UK

**Corresponding Author:**

Peter Moore, [ptmoorie@hotmail.com](mailto:ptmoorie@hotmail.com)

S2 Table: Parameters used for the scenario analyses

| **Parameter** | **Input value** | **Source** |
| --- | --- | --- |
| Treatment discontinuation: Exponential - lambda | –7.475 | Studies 140 and 142 [1] |
| Treatment discontinuation: Weibull - lambda | –6.250 | Studies 140 and 142 [1] |
| Treatment discontinuation: Weibull - gamma | –0.232 | Studies 140 and 142 [1] |
| Treatment discontinuation: Lognormal - lambda | 7.663 | Studies 140 and 142 [1] |
| Treatment discontinuation: Lognormal - sigma | 0.716 | Studies 140 and 142 [1] |
| Treatment discontinuation: Loglogistic - lambda | 7.559 | Studies 140 and 142 [1] |
| Treatment discontinuation: Loglogistic - gamma | 0.150 | Studies 140 and 142 [1] |
| TP - aboBoNT­A plus BSC - days coefficient (no transformation) - weighted | 0.0002 | Studies 140 and 142 [1] |
| TP - aboBoNT­A plus BSC - /cut1 coefficient (no transformation) - weighted | –3.9390 | Studies 140 and 142 [1] |
| TP - aboBoNT­A plus BSC - /cut2 coefficient (no transformation) - weighted | –2.3269 | Studies 140 and 142 [1] |
| TP - aboBoNT­A plus BSC - /cut3 coefficient (no transformation) - weighted | 1.9681 | Studies 140 and 142 [1] |
| TP - aboBoNT­A plus BSC - /cut4 coefficient (no transformation) - weighted | 3.1902 | Studies 140 and 142 [1] |
| TP - BSC - days coefficient (no transformation) - weighted | –0.0067 | Study 140 [1] |
| TP - BSC - /cut1 coefficient (no transformation) - weighted | –2.7996 | Study 140 [1] |
| TP - BSC - /cut2 coefficient (no transformation) - weighted | 1.6382 | Study 140 [1] |
| Proportion of non-responders at treatment cycle 1 | 71.4% | Studies 140 and 142 [1] |
| Proportion of non-responders at treatment cycle 2 | 50.5% | Studies 140 and 142 [1] |
| Proportion of non-responders at treatment cycle 3 | 36.7% | Studies 140 and 142 [1] |
| Physiotherapist responders proportion | 100.00% | Danchenko et al. 2022 [2] |
| Physiotherapist non-responders proportion | 100.00% | Danchenko et al. 2022 [2] |
| General practitioner responders proportion | 36.00% | Danchenko et al. 2022 [2] |
| General practitioner non-responders proportion | 36.00% | Danchenko et al. 2022 [2] |
| Specialist responders proportion | 91.00% | Danchenko et al. 2022 [2] |
| Specialist non-responders proportion | 91.00% | Danchenko et al. 2022 [2] |
| Orthotics responders proportion | 0.00% | Danchenko et al. 2022 [2] |
| Orthotics non-responders proportion | 0.00% | Danchenko et al. 2022 [2] |
| Pain clinic responders proportion | 9.10% | Danchenko et al. 2022 [2] |
| Pain clinic non-responders proportion | 9.10% | Danchenko et al. 2022 [2] |
| Psychologist responders proportion | 0.00% | Danchenko et al. 2022 [2] |
| Psychologist non-responders proportion | 0.00% | Danchenko et al. 2022 [2] |
| Movement disorder nurse responders proportion | 0.00% | Danchenko et al. 2022 [2] |
| Movement disorder nurse non-responders proportion | 0.00% | Danchenko et al. 2022 [2] |
| Speech and language therapist responders proportion | 0.00% | Danchenko et al. 2022 [2] |
| Speech and language therapist non-responders proportion | 0.00% | Danchenko et al. 2022 [2] |
| Occupational therapist responders proportion | 54.50% | Danchenko et al. 2022 [2] |
| Occupational therapist non-responders proportion | 54.50% | Danchenko et al. 2022 [2] |
| Acupuncturist responders proportion | 0.00% | Danchenko et al. 2022 [2] |
| Acupuncturist non-responders proportion | 0.00% | Danchenko et al. 2022 [2] |
| Nurse/home care responders proportion | 27.30% | Danchenko et al. 2022 [2] |
| Nurse/home care Non-responders proportion | 27.30% | Danchenko et al. 2022 [2] |
| Orthopaedic team responders proportion | 18.20% | Danchenko et al. 2022 [2] |
| Orthopaedic team non-responders proportion | 18.20% | Danchenko et al. 2022 [2] |
| Specialist nurse responders proportion | 0.00% | Danchenko et al. 2022 [2] |
| Specialist nurse non-responders proportion | 0.00% | Danchenko et al. 2022 [2] |
| Wheelchair services responders proportion | 9.10% | Danchenko et al. 2022 [2] |
| Wheelchair services non-responders proportion | 9.10% | Danchenko et al. 2022 [2] |
| Physiotherapist responders annual frequency | 6.00 | Danchenko et al. 2022 [2] |
| Physiotherapist non-responders annual frequency | 6.00 | Danchenko et al. 2022 [2] |
| General practitioner responders annual frequency | 1.50 | Danchenko et al. 2022 [2] |
| General practitioner non-responders annual frequency | 2.00 | Danchenko et al. 2022 [2] |
| Specialist responders annual frequency | 3.50 | Danchenko et al. 2022 [2] |
| Specialist non-responders annual frequency | 1.50 | Danchenko et al. 2022 [2] |
| Orthotics responders annual frequency | 0.00 | Danchenko et al. 2022 [2] |
| Orthotics non-responders annual frequency | 0.00 | Danchenko et al. 2022 [2] |
| Pain clinic responders annual frequency | 4.00 | Danchenko et al. 2022 [2] |
| Pain clinic non-responders annual frequency | 6.00 | Danchenko et al. 2022 [2] |
| Psychologist responders annual frequency | 0.00 | Danchenko et al. 2022 [2] |
| Psychologist non-responders annual frequency | 0.00 | Danchenko et al. 2022 [2] |
| Movement disorder nurse responders annual frequency | 0.00 | Danchenko et al. 2022 [2] |
| Movement disorder nurse non-responders annual frequency | 0.00 | Danchenko et al. 2022 [2] |
| Speech and language therapist responders annual frequency | 0.00 | Danchenko et al. 2022 [2] |
| Speech and language therapist non-responders annual frequency | 0.00 | Danchenko et al. 2022 [2] |
| Occupational therapist responders annual frequency | 2.00 | Danchenko et al. 2022 [2] |
| Occupational therapist non-responders annual frequency | 1.50 | Danchenko et al. 2022 [2] |
| Acupuncturist responders annual frequency | 0.00 | Danchenko et al. 2022 [2] |
| Acupuncturist non-responders annual frequency | 0.00 | Danchenko et al. 2022 [2] |
| Nurse/home care responders annual frequency | 65.00 | Danchenko et al. 2022 [2] |
| Nurse/home care non-responders annual frequency | 65.00 | Danchenko et al. 2022 [2] |
| Orthopaedic team responders annual frequency | 3.50 | Danchenko et al. 2022 [2] |
| Orthopaedic team non-responders annual frequency | 3.50 | Danchenko et al. 2022 [2] |
| Specialist nurse responders annual frequency | 0.00 | Danchenko et al. 2022 [2] |
| Specialist nurse non-responders annual frequency | 0.00 | Danchenko et al. 2022 [2] |
| Wheelchair services responders annual frequency | 1.00 | Danchenko et al. 2022 [2] |
| Wheelchair services non-responders annual frequency | 1.00 | Danchenko et al. 2022 [2] |
| Physiotherapist cost per visit | 65.85 | Medicare Benefits schedule (MBS). Code 10960. [3] |
| General practitioner cost per visit | 95.13 | Medicare Benefits schedule (MBS). Average value between codes 23,46 and 44. [3] |
| Specialist cost per visit | 134.70 | Medicare Benefits schedule (MBS). Code 107. [3] |
| Orthotics cost per visit | 119.30 | Medicare Benefits schedule (MBS). Code 47921. [3] |
| Pain clinic cost per visit | 90.45 | Kozor et al. 2022 [4] |
| Psychologist cost per visit | 105.45 | Medicare Benefits schedule (MBS). Code 80000. [3] |
| Movement disorder nurse cost per visit | 34.06 | Medicare Benefits schedule (MBS). Average value between codes 82200, 82205, 82210 and 82215. [3] |
| Speech and language therapist cost per visit | 92.95 | Medicare Benefits schedule (MBS). Code 93033. [3] |
| Occupational therapist cost per visit | 65.85 | Medicare Benefits schedule (MBS). Code 10958. [3] |
| Acupuncturist cost per visit | 57.48 | Medicare Benefits schedule (MBS). Average value between codes 193 and 197. [3] |
| Nurse/home care cost per visit | 34.06 | Medicare Benefits schedule (MBS). Average value between codes 82200, 82205, 82210 and 82215. [3] |
| Orthopaedic team cost per visit | 134.70 | Medicare Benefits schedule (MBS). Code 107. [3] |
| Specialist nurse cost per visit | 34.06 | Medicare Benefits schedule (MBS). Average value between codes 82200, 82205, 82210 and 82215. [3] |
| Wheelchair services cost per visit | 500.00 | Push Mobility 2022. [5] |

Abbreviations: aboBoNT­A, abobotulinumtoxinA; AUD, Australian dollar; BSC, best supportive care; MBS, Medicare Benefits Schedule; PBS DPMQ, Pharmaceutical Benefits Scheme’s Dispensed Price for Maximum Quantity; TP, transition probability.

References

1. Gracies JM, Esquenazi A, Brashear A, Banach M, Kocer S, Jech R, et al. Efficacy and safety of abobotulinumtoxinA in spastic lower limb: Randomized trial and extension. Neurology. 2017 Nov 28;89(22):2245-53.

2. Danchenko N, Johnston KM, Whalen J. The cost-effectiveness of abobotulinumtoxinA (Dysport) and onabotulinumtoxinA (Botox) for managing spasticity of the upper and lower limbs, and cervical dystonia. J Med Econ. 2022 Jan-Dec;25(1):919-29.

3. Medicare Benefits Schedule (MBS). Benefit codes. Available at http://www9.health.gov.au/mbs/search.cfm. Accesesd 02 December 2022.

4. Kozor R, Mooney J, Lowe H, Kritharides L, Altman M, Klimis H, et al. Rapid Access Chest Pain Clinics: An Australian Cost-Benefit Study. Heart, Lung and Circulation. 2022 2022/02/01/;31(2):177-82.

5. Push Mobility. Repairs and maintenance. Available at https://www.pushmobility.com.au/pages/repairs-maintenance. Accessed 02 December 2022.
